# Advances in targeted therapies and new promising targets in esophageal cancer

**DOI:** 10.18632/oncotarget.2752

**Published:** 2015-01-16

**Authors:** Abbes Belkhiri, Wael El-Rifai

**Affiliations:** ^1^ Department of Surgery, Vanderbilt University Medical Center, Nashville, Tennessee 37232, USA; ^2^ Department of Veterans Affairs, Tennessee Valley Healthcare System, Nashville, Tennessee 37212, USA

**Keywords:** adenocarcinoma, esophageal, gastric, cancer, targeted therapy

## Abstract

Esophageal cancer, comprising squamous carcinoma and adenocarcinoma, is a leading cause of cancer-related death in the world. Notably, the incidence of esophageal adenocarcinoma has increased at an alarming rate in the Western world. Unfortunately, the standard first-line chemo-radiotherapeutic approaches are toxic and of limited efficacy in the treatment of a significant number of cancer patients. The molecular analysis of cancer cells has uncovered key genetic and epigenetic alterations underlying the development and progression of tumors. These discoveries have paved the way for the emergence of targeted therapy approaches. This review will highlight recent progress in the development of targeted therapies in esophageal cancer. This will include a review of drugs targeting receptor tyrosine kinases and other kinases in esophageal cancer. Additional studies will be required to develop a rational integration of these targeted agents with respect to histologic types of esophageal cancer and the optimal selection of cancer patients who would most likely benefit from targeted therapy. Identification of AURKA and AXL as key molecular players in esophageal tumorigenesis and drug resistance strongly justifies the evaluation of the available drugs against these targets in clinical trials.

## INTRODUCTION

The increasing incidence and poor prognosis of esophageal cancer represent a major public health problem worldwide. In 2013, it was estimated that 17,990 new cases of esophageal cancer will be diagnosed and only 15% of patients will survive their disease in the United States [1]. This malignancy comprises two major histologic types, esophageal squamous cell carcinoma (SCC) and esophageal adenocarcinoma (AC)—together they account for the sixth leading cause of death in the world [2]. Although in the last few decades SCC cases have steadily decreased, the incidence of AC has increased at an alarming rate (> 6-fold) in the Western world [3]. SCC and AC differ substantially in their underlying etiology factors and tumorigenesis. While smoking and alcohol [4], prior head and neck cancer [5], and human papilloma virus infection [6] are risk factors in SCC; gastro-esophageal reflux disease (GERD) and obesity have been associated with increased risk of AC [7]. SCC develops from a premalignant dysplastic lesion that originates from the native squamous epithelium, whereas the development of AC is initiated from an intestinal metaplastic lesion (Barrett's esophagus, BE) that occurs in response to GERD [8]. In addition to surgical resection, the current standard of care for patients with either SCC or AC is chemotherapy with cisplatin and 5-fluorouracil (5-FU) [9], and in combination with other agents such as oxaliplatin [10] and irinotecan [11]. Unfortunately, the majority of patients at advanced stages of the disease fail to benefit from these treatments as the 5-year survival rate remains < 15% [12], underscoring the critical need for more effective therapies. Hence, there is an urgent necessity to identify the underlying molecular alterations of SCC and AC, and characterize molecular signatures to distinguish the two types of esophageal cancer. Genomic, proteomic, and molecular epidemiologic studies have greatly helped identify potential therapeutic targets that could eventually overcome the shortcomings of the current standard therapies of esophageal cancer. As part of the International Cancer Genome Consortium project, Song and colleagues [13] conducted a comprehensive genomic analysis of SCC samples and identified several significantly mutated genes, among which *ADAM29* and *FAM135B*. These two genes have not been previously described in SCC. Of note, *FAM135B* has been characterized as a novel cancer gene that promotes malignancy of SCC cells [13]. A recent similar study on AC samples revealed many new significantly mutated genes including *DOCK2* and *ELMO1* [14]. Functional analysis indicated that mutations in *ELMO1*, found in EAC, significantly enhanced cellular invasion; this suggests the potential contribution of RAC1 signaling to Barrett's tumorigenesis [14]. These studies provide a foundation for further investigations to characterize subsets of esophageal cancer that may be clinically useful for developing more effective therapies. Based on the current literature, this review summarizes the targeted therapies in clinical development and proposes potential novel therapeutic targets in esophageal SCC and AC.

### Targeted therapies for esophageal cancer in development

During the past two decades, the emerging understanding of the underlying molecular mechanisms of carcinogenesis that helped identify important molecular targets led to the development of drugs that target specific molecular sites for various types of cancer, including esophageal SCC and AC. These drugs inhibit or interfere with key molecules or signaling pathways that regulate cell growth and proliferation, angiogenesis, apoptosis, invasion and metastasis, and inflammation. Recently, some of these drugs have been clinically tested as monotherapy or in combination with chemotherapy and/or radiotherapy on esophageal and gastro-esophageal junction cancers (Table 1).

**Table 1:** Selected ongoing and recent clinical studies of targeted agents in esophageal and gastro-esophageal junction cancers (ClinicalTrials.gov)

Molecular target	Agent	Histology (number of enrolled patients)	Monotherapy (NCT identifier, clinical trial phase)	Agent + chemotherapy and/or radiotherapy (NCT identifier, clinical trial phase)
EGFR	Nimotuzumab	SCC (20)	NCT02011594, P2	
		SCC (9, 144)	NCT01993784, P1/2	NCT01232374, P2
		SCC/AC (104)		NCT01249352, P2/3
	Panitumumab	SSC (300)		NCT01627379, P3
		SSC/AC (36)		NCT01128387, P1/2
		AC/GEJ (574)		NCT00824785, P3
	Cetuximab	GEJ (904)		NCT00678535, P3
	Icotinib	SCC (50)	NCT01973725, P2	
	Gefitinib	AC/GEJ (72)	NCT00100945, P2	
		SCC/AC (70)		NCT01291823, P2
		SCC/AC/GEJ (80)		NCT00258323, P2
VEGFR-2	Ramucirumab	AC/GEJ (162)		NCT01246960, P2
		GEJ (665)		NCT01170663, P3
HER-2	Pertuzumab/Trastuzumab	GEJ (780)		NCT01774786, P3
EGFR/HER-2	Lapatinib	SCC (24)	NCT01666431, P2	
		GEJ (28)		NCT00313599, P1
		AC/GEJ (13)		NCT01395537, P1/2
HER-2	Afatinib	GEJ (40)	NCT01522768, P2	
c-MET	Onartuzumab	GEJ (564)		NCT01662869, P3
	Rilotumumab	GEJ (450)		NCT02137343, P3
RTKs	Sunitinib	GEJ (98)		NCT00891878, P2
		GEJ (30)		NCT00524186, P1
	Sorafenib	SCC/AC/GEJ (35)	NCT00917462, P2	
Src/Abl	Saracatinib	GEJ (21)	NCT00607594, P2	
AURKA	MLN8237	GEJ (273)	NCT01045421, P1/2	
AKT	MK2206	GEJ (75)	NCT01260701, P2	
PI3K	BKM-120	SCC (41)	NCT01806649, P2	
mTOR	RAD001	SCC/AC (50)	NCT00985192, P2	
		AC/GEJ (44)		NCT01231399, P1/2
		SCC/AC (52)		NCT01490749, P1
Proteasome	Bortezomib	GEJ (58)		NCT00061932, P2

Abl, Abelson murine leukemia; AC, esophageal adenocarcinoma; AURKA, aurora kinase A; EGFR, epidermal growth factor receptor; GEJ, gastro-esophageal junction adenocarcinoma; HER-2, human epidermal growth factor receptor; mTOR, mammalian target of rapamycin; PI3K, phosphoinositide 3-kinase; RTKs, receptor tyrosine kinases; SCC, esophageal squamous cell carcinoma; Src, Rous sarcoma virus tyrosine kinase homolog; VEGFR, vascular endothelial growth factor receptor; P, clinical trial phase.

### Receptor tyrosine kinases

**Figure 1:**
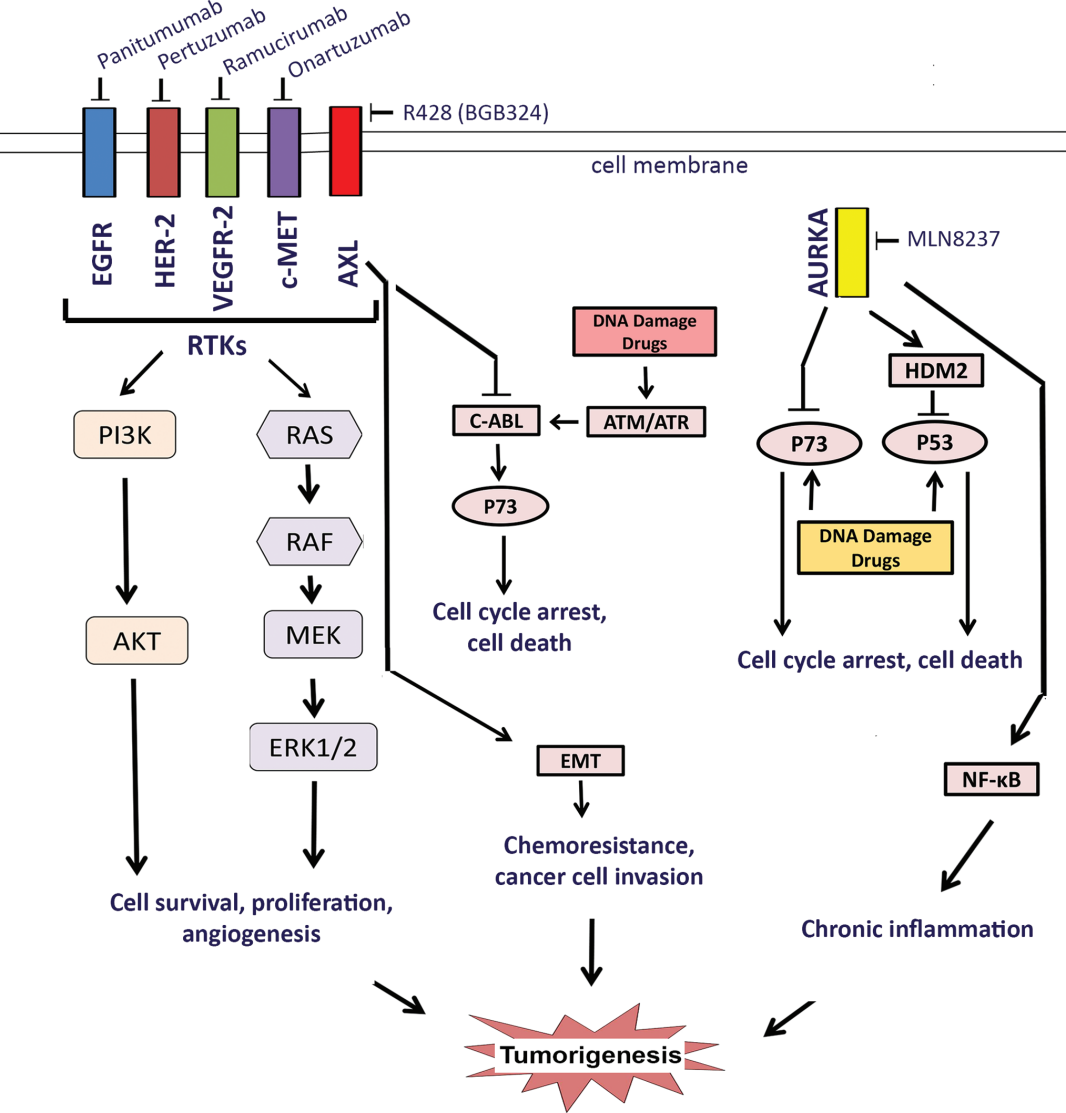
Constitutive activation of receptor and non-receptor protein kinases signaling promotes esophageal and gastro-esophageal tumorigenesis Overexpression and/or gene amplification of RTKs, and to a lesser extent gain-of-function mutations of RTKs, lead to constitutive activation of down-stream signaling, resulting in uncontrolled cell growth, cell survival, angiogenesis, and ultimately tumorigenesis. Of note, AXL suppresses DNA damage-induced apoptosis through interaction and inhibition of c-ABL tyrosine kinase in esophageal AC [64]. In addition, AXL has been implicated in promoting epithelial-to-mesenchymal transition (EMT), cancer cell invasion, and drug resistance in many types of malignancies, including those of the breast [68, 90] and lung [72, 91]. Overexpression of AURKA, a serine/threonine kinase, attenuates DNA damage-induced cell cycle arrest and apoptosis through inhibition of p53 family members [84, 89]. Furthermore, AURKA has been shown to activate NF-κB signaling, leading to chronic inflammation and gastric tumorigenesis [92]. Targeted monoclonal antibodies, panitumumab (EGFR), pertuzumab (HER-2), ramucirumab (VEGFR-2), and onartuzumab (c-MET), are currently being tested in combination with first-line chemotherapies in patients with gastric and gastro-esophageal cancers (phase III clinical trials, Table 1). The AXL inhibitor, R428 (BGB324), AURKA inhibitor, MLN8237, have been investigated in esophageal AC, mostly in pre-clinical studies. However, a limited phase I/II clinical study has tested MLN8237 in patients with solid tumors including esophageal cancers (results not reported).

Receptor tyrosine kinases (RTKs) are the main mediators that transduce extracellular signals into the cell to modulate cellular growth and differentiation. A large body of evidence indicating that the normally tightly controlled and regulated RTKs in normal cells are overexpressed, amplified, and mutated, leading to constitutive activation of RTK signaling, resulting in deregulated cell growth and tumorigenesis (reviewed by [15]). Similarly, gain-of-function mutations of RTK downstream effectors such as RAS, RAF, and PI3K can promote cancer (reviewed by [16]). Our increasing knowledge of the mechanisms of uncontrolled RTK signaling in cancer has provided the rationale for the development of drugs against RTK signaling components (Figure 1).

### Epidermal growth factor receptor

The human epidermal growth factor receptor (EGFR), an HER family member, is a 170-kDa protein with an extracellular ligand-binding domain, a transmembrane domain, and a cytosolic tyrosine kinase domain [17]. Epidermal growth factor and transforming growth factor-α ligands bind and activate the EGFR receptor, followed by internalization of homodimerized or heterodimerized (EGFR binding HER-2 or ERBB3) receptors, leading to autophosphorylation of the intracellular tyrosine kinase domain and activation of several downstream pro-oncogenic signaling pathways [18]. Based on its aberrant activation as a result of gene amplification, overexpression and mutation, and its association with tumorigenesis and poor prognosis; EGFR has been targeted by two classes of drugs: 1) anti-EGFR monoclonal antibodies (mAb) directed at the extracellular domain of the receptor and 2) small molecule EGFR tyrosine kinase inhibitors (TKI). Targeting RGFR with mAbs either as monotherapy or in combination with conventional chemotherapy has proven its first clinical success in patients with advanced colorectal cancer, especially those with wild-type *KRAS* [19, 20]. EGFR mutations (5–10%) [21], amplification (20–30%), and overexpression (30–80%) in human esophageal SCC and AC have provided the rationale for targeting EGFR in esophageal cancer [22]. This suggests that EGFR amplification and overexpression rather than mutations drive esophageal cancers. Cetuximab, which is a humanized mouse EGFR mAb, has been shown to downregulate EGF-induced EGFR phosphorylation, inhibit homodimerization and heterodimerization of EGFR with HER-2 and downstream signaling in preclinical cell models of gastro-esophageal cancer [23]. In phase II clinical studies, cetuximab in combination with standard chemotherapy regimens significantly improved response rates in patients with gastro-esophageal junction (GEJ) cancer [24] or SCC [25]. However, cetuximab as a single agent has little clinical activity in upper gastrointestinal cancers [26]. A recent randomized phase III clinical study concluded that the combination of cetuximab with capecitabine and cisplatin had no additional clinical advantage to chemotherapy alone in GEJ cancer patients [27]. Nimotuzumab, a humanized EGFR mAb, in combination with standard chemotherapy (cisplatin or 5-fluorouracil) has shown good therapeutic response in patients with SCC [28]. Another EGFR mAb, panitumumab, has been tested in combination with epirubicin, oxaliplatin and capecitabine in metastatic GEJ cancer patients in a randomized phase III clinical trial [29]. The results from this study indicated that addition of panitumumab to the chemotherapy does not increase overall survival.

Several TKIs targeting EGFR have been clinically tested in upper gastrointestinal cancers. In a phase II study, gefitinib in combination with radiotherapy and chemotherapy in patients with advanced esophageal SCC or AC enhanced overall survival [30]. However, in another phase II clinical trial, gefitinib alone has shown very minimal clinical activity in patients with esophageal SCC/AC and GEJ, suggesting that better patient selection and combination with chemotherapy regimens may enhance the clinical outcome [31]. In a phase II clinical trial, erlotinib (TKI) in combination with concurrent chemotherapy and radiotherapy in patients with advanced esophageal carcinomas has significantly improved the overall clinical response [32]. In a separate phase II study, erlotinib as a single agent in patients with unresectable or metastatic GEJ adenocarcinoma has shown some clinical benefits [33].

### Human epidermal growth factor receptor 2

The human epidermal growth factor receptor 2 (HER-2), a member of the HER family, is a 185-kDa transmembrane RTK without a known activating ligand [34]. HER-2 is activated through its dimerization with other members of the HER family including EGFR and HER-3 [35], leading to the subsequent activation of downstream signaling. The fact that HER-2 overexpression and amplification have been associated with poor prognosis in ovarian and breast cancers [36, 37], led to the development and approval of trastuzumab mAb to target HER-2 in breast tumors [38]. The initial success of trastuzumab targeted therapy in breast cancer led to its investigation in other types of HER- 2-overexpressing cancers. Overexpression of HER-2 in esophageal SCC (23%) and GEJ (22%) adenocarcinomas have been associated with poor response to neoadjuvant chemotherapy and overall poor survival, respectively [39]. In a Japanese clinical study, trastuzumab in combination with capecitabine/cisplatin or 5-fluorouracil/cisplatin in patients with advanced GEJ cancer improved overall survival as compared to chemotherapy alone [40]. In a randomized phase III study, pertuzumab mAb, which is directed against HER-2, in combination with trastuzumab, 5-fluorouracil, capecitabine, and cisplatin is currently investigated in patients with HER-2-positive metastatic gastric or GEJ adenocarcinoma (NCT01774786). In addition, a randomized phase III clinical trial is currently ongoing to evaluate the efficacy and safety of trastuzumab emtansine (T-DM1) compared to standard taxane treatment in patients with HER-2-positive advanced or metastatic gastric or GEJ adenocarcinoma (NCT01641939). Lapatinib (TKI), a small-molecule inhibitor, which targets both EGFR and HER-2, was effective in patients with trastuzumab-resistant breast cancer [41]. In a phase II clinical study, lapatinib alone in patients with EGFR- and/or HER-2-overexpressing esophageal AC showed very minimal clinical benefits [42]. In addition, a phase I clinical trial is currently ongoing to evaluate lapatinib in combination with paclitaxel in patients with GEJ adenocarcinoma (NCT00313599).

### Mesenchymal-epithelial transition factor

The mesenchymal-epithelial transition factor (c-MET) RTK, consisting of alpha subunit (45 kDa) and beta subunit (145 kDa), is activated by its ligand hepatocyte growth factor (HGF), leading to transduction of downstream signaling, including PI3K/AKT, mTOR, and STAT3 pathways [43]. Physiologically, c-MET promotes cell scattering and motility, which involves the disruption of cell-cell adhesion—a critical function in embryogenesis and wound healing [44]. The MET pathway has been chosen as a promising drug target because dysregulation of this pathway occurs in various types of human cancers, including human gastric and esophageal AC [45]. Several molecular mechanisms such as ligand/receptor overexpression or *c*-*MET* gene amplification can mediate such dysregulation. Notably, gene amplification of *c-MET* has been associated with poor prognosis in gastric and esophageal cancers [46]. The c-MET and its ligand HGF are potential therapeutic targets in upper gastrointestinal cancers. For instance, in a randomized global phase III study, onartuzumab mAb (MetMAb), which is directed against c-MET receptor, is currently investigated in combination with 5-fluorouracil, folinic acid, and oxaliplatin (mFOLFOX6) in patients with metastatic HER2-negative and MET-positive gastric and GEJ adenocarcinoma (NCT01662869). In addition, crizotinib, which is a small molecule inhibitor of c-MET—approved for the treatment of ALK-positive NSCLC by the FDA, has been shown to be effective in the treatment of a subset of patients with *c-MET*-amplified gastric or esophageal tumors [47]. Another c-MET TKI, tivantinib, is currently being tested in combination with chemotherapy (FOLFOX) in patients with GEJ and gastric adenocarcinoma (NCT01611857). The HGF mAb, rilotumumab, in combination with chemotherapy (ECX) has shown some survival benefit in patients with gastric and GEJ cancers, especially those expressing higher levels of c-MET [48]. Previous studies showed that acquired resistance to EGFR inhibitors was associated with gene amplification of *c-MET* in NSCLC [49], suggesting that combined targeting of EGFR and c-MET could be beneficial to overcome the onset of drug resistance.

### Vascular endothelial growth factor

The vascular endothelial growth factor (VEGF), which belongs to the platelet-derived growth factor family, is the most potent proangiogenic factor that interacts with its receptors (VEGFR-1, -2, and -3). This will ultimately lead to vasculogenesis and angiogenesis [50]. Frequent overexpression of VEGF has been associated with increased microvessel density, tumor invasion and metastasis, and poor prognosis in many types of cancers [51]. VEGF is up-regulated in BE and esophageal AC, and overexpression of VEGF protein has been reported as a potential negative prognostic marker in esophageal SCC [51]. Therefore, VEGF may be a potential therapeutic target in esophageal cancers. In a phase III study, bevacizumab mAb, which binds to all isoforms of VEGF and hence prevent its binding to its receptors, in combination with chemotherapy (irinotecan) improved the overall response rate and survival of patients with colorectal carcinoma [52]. A phase II study revealed that bevacizumab in combination with irinotecan and cisplatin relatively enhanced the survival and clinical outcome of patients with metastatic gastric or GEJ adenocarcinoma [53]. Similarly, in a more recent phase II clinical trial, the combination of bevacizumab with docetaxel, cisplatin, and fluorouracil improved response rate and survival as compared to chemotherapy alone in patients with metastatic GEJ adenocarcinoma [54]. Ramucirumab, a fully humanized monoclonal antibody that binds to VEGFR2 and blocks angiogenesis, has been recently approved by the FDA as second-line therapy for patients with advanced gastric cancer or GEJ adenocarcinoma [55]. In a randomized phase III trial, ramucirumab monotherapy has improved survival in patients with advanced gastric or GEJ adenocarcinoma after first-line chemotherapy [56]. Several TKIs, which inhibit multiple RTKs including VEGF receptor, have been investigated. Sunitinib, as a single agent, has insufficient clinical value in patients with gastric or GEJ adenocarcinoma [57]. In addition, sunitinib is currently investigated in combination with chemotherapy (FOLFIRI) in patients with gastric and esophageal cancers (NCT00891878, NCT00524186). Another TKI, sorafenib, in combination with docetaxel and cisplatin improved the overall survival in patients with metastatic gastric or GEJ adenocarcinoma [58]. However, in a phase II study, sorafenib as a single agent is being investigated in patients with metastatic or recurrent esophageal SCC and AC, and GEJ adenocarcinoma (NCT00917462).

### AXL

AXL, a receptor tyrosine kinase that belongs to the TAM subfamily (Tyro-3, AXL, and Mer), is a transmembrane protein that consists of extracellular two immunoglobin-like domains with two tandem fibronectin type III repeats, and a cytoplasmic kinase domain [59]. AXL binds to its ligand Gas-6 (growth arrest-specific gene 6) and activates downstream signaling, including PI3K/AKT and NF-κB pathways [60]. Following its identification as a transforming gene in human chronic myelogenous leukemia [61], AXL has been shown to be overexpressed in several types of human cancers such as lung [62] and gastric [63] cancers. Of note, we [64], and others [65], have reported AXL overexpression in esophageal AC. In fact, AXL overexpression has been associated with tumor cell migration and invasion, epithelial-to-mesenchymal transition (EMT), and poor survival in different types of cancers [66, 67]. These studies highlight AXL as a promising target for cancer therapy. In this regard, Holland and colleagues [68] generated and characterized a highly selective small-molecule inhibitor (R428, Rigel Pharmaceuticals) that potently blocks the catalytic activity of AXL and AXL-dependent biological functions, including tumor cell invasion and angiogenesis in tumor mouse models. In a preclinical study, Li and colleagues [69] have developed AXL monoclonal antibodies that suppress NSCLC xenograft growth through downregulation of AXL expression and induction of apoptosis. Preclinical studies on AXL inhibitor TP-0903 (Tolero Pharmaceuticals & Astex Pharmaceuticals) in pancreatic and lung cancers are ongoing [70]. BergenBio & Rigel Pharmaceuticals have recently launched a clinical trial (phase I) to evaluate the AXL inhibitor BGB324 (formerly R428) in cancer patients [70]. In another phase I clinical study, a compound (S49076, Servier) that inhibits AXL/Mer and fibroblast growth factor receptors has been evaluated in patients with advanced solid tumors [70]. However, a new study urges caution as systemic inhibition of AXL and its closely related RTK, Mer, could promote colon cancers [71]. Of note, AXL and Mer are expressed in macrophages and dendritic cells mediating anti-inflammatory functions that prevent colon tumorigenesis [71]. Therefore, targeting AXL with the highly specific AXL inhibitors may be a less risky therapeutic approach than using other inhibitors with broad range specificity. The fact that AXL is frequently overexpressed and associated with poor prognosis in esophageal AC strongly suggests that AXL is an attractive therapeutic target in this human malignancy.

A recent interesting study demonstrated that activation of AXL kinase mediates resistance to EGFR-targeted therapy in lung cancer [72]. Another study showed that knockdown of AXL significantly improved *in vitro* response to standard chemotherapy by promoting apoptosis in NSCLC [73]. In this regard, we investigated the potential role of AXL in promoting resistance to standard chemotherapeutic agents in esophageal AC. Indeed, we demonstrated that AXL mediates resistance to the DNA-damaging drug (cisplatin) through regulation of c-ABL tyrosine kinase in p53-deficient esophageal AC cells. Mechanistic investigations showed that AXL attenuates cisplatin-induced apoptosis through blocking nuclear accumulation of c-ABL and phosphorylation of p73 in response to DNA damage (Figure 1) [64]. The findings from this study and others [65] strongly support an initiative to evaluate AXL as a therapeutic target in combination with DNA-damaging chemotherapeutic agents in esophageal AC in preclinical and clinical settings. This combination therapeutic approach may be valuable to overcome the frequent occurrence of drug resistance in patients with esophageal AC.

### Aurora kinase A

Aurora kinase A (AURKA)—a member of a family that also includes aurora kinase B and C—is a serine/threonine kinase, which is crucial for normal mitotic spindle formation and centrosome maturation and separation during cell division [74]. AURKA kinase activity is dependent on the phosphorylation of a threonine residue (T288) located on the activation loop of the kinase. We, and others, have previously reported the amplification of the region band 13, which harbors the *AURKA* gene, on the long arm of chromosome 20 (20q13) in upper gastrointestinal cancers [75, 76]. In addition, the *AURKA* gene is frequently amplified and/or overexpressed in several types of cancer including those of the stomach [77] and esophagus [78]. Notably, AURKA overexpression has been significantly associated with more advanced stages of cancer and poor prognosis [79]. Pre-clinical studies showed that activation of AURKA leads to transformation of rodent fibroblast cells and the formation of multipolar mitotic spindles that induce genomic instability [80], establishing AURKA as a bona fide oncogene. AURKA blocks p53 tumor suppressor function through direct phosphorylation of p53 at Ser315, inducing MDM-2 mediated degradation of p53 protein [81]; and Ser215 to suppress its transcriptional activity in cancer cells [82]. Additionally, we uncovered that AURKA suppresses p53 through regulation of AKT-MDM-2 signaling in gastric cancer cells [83], and attenuates TAp73 transcriptional activity in p53 deficient cancer cells (Figure 1) [84]. We [83], and others [85], have reported that AURKA promotes survival of cancer cells through increased phosphorylation of AKT at Ser473. Because of their overexpression and association with tumorigenesis, aurora kinase family members have become the focus of drug discovery. Several aurora kinase small molecule inhibitors have been reviewed by Pollard and colleagues [86]. An increasing number of these inhibitors have been developed at preclinical or clinical stages. A second-generation investigational AURKA inhibitor (MLN8237/alisertib, Millennium), which has 200-fold selectivity for AURKA over AURKB (aurora kinase B) in cell assays, has been recently investigated as monotherapy in phase I clinical trials in patients with advanced solid tumors or lymphomas (NCT01898078, NCT00962091). Notably, a phase I/II study on MLN8237 in patients with solid tumors including esophageal and gastric cancers has just been completed, yet the results have not been reported to date (NCT01045421). In preclinical studies, we investigated whether the combination of MLN8237 with standard chemotherapeutic agents could have a better therapeutic outcome in upper gastrointestinal cancers. Indeed, we found that MLN8237 in combination with cisplatin or docetaxel significantly enhanced cell death in esophageal AC xenograft mouse models [87, 88]. Mechanistic investigations indicated that AURKA promotes resistance to DNA damaging agents through activation of HDM2, a negative regulator of p53, thereby confirming that inhibition of AURKA in combination with chemotherapy is a good therapeutic approach in upper gastrointestinal cancers [89]. These results strongly support the initiation of clinical studies on MLN8237 in combination with standard chemotherapeutic agents in patients with upper gastrointestinal cancers. Additional preclinical studies on combinations of MLN8237 with targeted drugs (listed above) are required prior to designing clinical trials in patients with upper gastrointestinal cancers, especially those who have developed acquired resistance to first-line chemotherapy.

### Conclusions

Esophageal cancer is characterized by resistance to current first-line therapies and dismal clinical outcome, underscoring the urgent need for more effective and innovative treatment strategies. Unlike other types of cancer, elucidation of the molecular mechanisms that regulate esophageal tumorigenesis of both histologic types has been incomplete. Our understanding of cancer biology coupled with the development of targeted drugs will have a positive impact in the treatment of esophageal cancer. The current ongoing clinical trials of targeted drugs in esophageal cancer patients have been mostly based on molecular targets identified in other malignancies. However, identification of AURKA and AXL as important players in esophageal tumorigenesis and drug resistance strongly justifies evaluating the available drugs targeting these molecules in clinical trials. As targeted therapy has been marred by drug resistance, it is imperative to preemptively identify the underlying molecular mechanisms and develop strategies to overcome them in a clinical setting. Notably, development of biomarkers of drug response and resistance may be useful in future clinical trials and rational management of cancer patients.
